# Multiple detection and spread of novel strains of the SARS-CoV-2 B.1.177 (B.1.177.75) lineage that test negative by a commercially available nucleocapsid gene real-time RT-PCR

**DOI:** 10.1080/22221751.2021.1933609

**Published:** 2021-06-13

**Authors:** Laura Amato, Lucija Jurisic, Ilaria Puglia, Valeria Di Lollo, Valentina Curini, Giuseppe Torzi, Arturo Di Girolamo, Iolanda Mangone, Adamo Mancinelli, Nicola Decaro, Paolo Calistri, Francesca Di Giallonardo, Alessio Lorusso, Nicola D’Alterio

**Affiliations:** a Istituto Zooprofilattico Sperimentale dell’Abruzzo e Molise (IZSAM), Teramo, Italy; b Faculty of Veterinary Medicine, Università degli Studi di Teramo, Teramo, Italy; c Azienda Sanitaria Locale, Lanciano-Vasto-Chieti, Chieti, Italy; d Department of Veterinary Medicine, University of Bari Aldo Moro, Valenzano (Bari), Italy; e The Kirby Institute, The University of New South Wales (UNSW), Sydney, Australia

**Keywords:** Abruzzo, COVID-19, diagnosis, mutations, nucleocapsid, polymerase chain reaction, SARS-CoV-2, epidemiology

## Abstract

Several lineages of SARS-CoV-2 are currently circulating worldwide. During SARS-CoV-2 diagnostic activities performed in Abruzzo region (central Italy) several strains belonging to the B.1.177.75 lineage tested negative for the N gene but positive for the ORF1ab and S genes (+/+/- pattern) by the TaqPath COVID-19 CE-IVD RT-PCR Kit manufactured by Thermofisher. By sequencing, a unique mutation, synonymous 28948C > T, was found in the N-negative B.1.177.75 strains. Although we do not have any knowledge upon the nucleotide sequences of the primers and probe adopted by this kit, it is likely that N gene dropout only occurs when 28948C > T is coupled with 28932C > T, this latter present, in turn, in all B.1.177.75 sequences available on public databases. Furthermore, epidemiological analysis was also performed. The majority of the N-negative B.1.177.75 cases belonged to two clusters apparently unrelated to each other and both clusters involved young people. However, the phylogeny for sequences containing the +/+/- pattern strongly supports a genetic connection and one common source for both clusters. Though, genetic comparison suggests a connection rather than indicating the independent emergence of the same mutation in two apparently unrelated clusters. This study highlights once more the importance of sharing genomic data to link apparently unrelated epidemiological clusters and to, remarkably, update molecular tests.

## Introduction

Hallmark of coronaviruses (CoVs) is their exceptional genetic plasticity which may promote changes in their antigenic profile, tissue tropism or host range by means of two distinct mechanisms. First, the viral replicase (an RNA dependent-RNA polymerase) does not possess a good proof-reading activity, therefore the incorporation of wrong nucleotides at each replication cycle and the consequent accumulation of mutations in the viral genome lead to a progressive differentiation of the viral progeny from the parental strain. This mechanism may cause the progressive adaptation of the viral surface proteins to the cell receptors, thus increasing the viral fitness. Second, the distinctive replicating machinery of CoVs facilitates homologous and heterologous recombination events [1,2].

Severe acute respiratory syndrome coronavirus 2 (SARS-CoV-2) (species *Severe acute respiratory syndrome-related coronavirus*, subgenus *Sarbecovirus*, genus *Betacoronavirus*, family *Coronaviridae*) is the causative agent of the current pandemic of CoV respiratory disease, named by the WHO coronavirus disease 2019 (COVID-19) [3–5]. Since its emergence, SARS-CoV-2 evolved rapidly and several viral lineages (containing unique constellations of mutations, including several of known biological importance located at the S1 portion of the spike protein) are now circulating worldwide [6]. Within these lineages, B.1.1.7, P.1, and B.1.351 gained international concern as for their enhanced transmission capabilities, mortality rates and/or reduced neutralization of specific immunity stimulated by previous infections or by vaccines against them [7].

However, besides being able to affect viral pathobiology [8–11], mutations may have significant impact on virus direct diagnosis by polymerase chain reaction (PCR)-based assays. Fast and accurate molecular diagnosis of SARS-CoV-2 RNA out of naso-oropharingeal swab specimens is the first step to quickly identify positive cases, and thus, preserve the virus from further spreading and limiting the impact of the pandemic in the human population. Hence, mutations occurring in those regions of the genome that are the target of molecular tests may generate false negative results, which could hamper proper diagnosis, contact tracing, and quarantine measures.

Several real-time RT-PCR assays have been developed and are commercially available for detection of SARS-CoV-2 RNA, which are generally established to detect multiple (up to three) SARS-CoV-2 ORFs including the large viral polymerase gene located at the 5′-most two thirds of the SARS-CoV-2 genome and ORFs encoding for structural proteins located at the 3′-end of the genome. One of the most common kit for SARS-CoV-2 RNA diagnosis is the TaqPath COVID-19 CE-IVD RT-PCR Kit manufactured by Thermofisher^®^ (Thermo Fisher Scientific, Waltham, MA, USA). This test is able to detect simultaneously three different portions of the SARS-CoV-2 genome located in the ORF1ab, spike (S) and nucleocapsid (N) protein ORFs, respectively. This test is currently used for molecular diagnosis of SARS-CoV-2 at Istituto Zooprofilattico Sperimentale dell’Abruzzo e del Molise (IZSAM), a veterinary public health institute, designated by the Italian Ministry of Health and the Abruzzo region as diagnostic hub for COVID-19 diagnosis and genome analysis [12].

In this manuscript, we describe the detection of multiple strains belonging to the B.1.177.75 lineage which tested negative for the N gene with the Thermofisher molecular test but positive for the ORF1ab and S genes. The implications for SARS-CoV-2 diagnosis of mutations in the N gene, potentially involved in the failed detection of N target, are discussed.

## Materials and methods

### Ethical approval

The results analysed in the present study derive from the official control activities performed by the Public Health Local Authority of Abruzzo region and no ethical approval is specifically requested.

### Surveillance and epidemiological analysis

Specimens that are N-negative but positive for the ORF1ab and S (+/+/-) genes are considered positive for SARS-CoV-2 according to the manufacturer’s recommendations. The first three samples (2021TE101854, 2021TE101848 and 2021TE101850) showing this pattern were collected on 19 February 2021 in Torino di Sangro (Chieti province). Additional information was provided from the Local Health Authority as the three samples belonged to individuals of the same family. As a consequence, we monitored the spread of N-negative SARS-CoV-2 strains in a time period comprised between 20 February and 6 March 2021. N-gene negative samples were processed by means of an additional real-time RT-PCR targeting the N gene, 2019- nCoV_N1 [13]. Epidemiological analysis was performed for samples showing the +/+/- pattern supported by the Local Health Authority (Azienda Sanitaria Locale - ASL) as this later provided personal data and epidemiological information of the detected cases.

### Sequencing and genome analysis

Within the N-negative samples, those showing cycle threshold (*C_T_*) values <20 by the ORF1ab and S real-time RT-PCR assays, when possible, were further processed for whole genome sequencing by next generation sequencing (NGS) and for genome analysis as described previously by our group [12]. Samples were processed by NGS by means of two different protocols. The first included the Artic v3 protocol, whereas the second was based on the CovidSeq protocol. As for the Artic v3 protocol [14], cDNA was synthesized starting from 2.25 µl of purified RNA and then amplified with Q5 high-fidelity DNA polymerase (NEB) using each of two Artic v3 primer pools tiling the SARS-CoV-2 genome. Library preparation was carried out using Nextera DNA Flex Library Prep (Illumina Inc., San Diego, CA USA) and deep sequencing was performed on the MiniSeq (Illumina Inc., San Diego, CA, USA) by the MiniSeq Mid Output Kit (300-cycles) and standard 150 bp paired-end reads. As for the CovidSeq protocol, the libraries were prepared from 8.5 µl of purified RNA, according to the manufacturer’s protocol (Illumina COVIDSeq Test, Illumina Inc, San Diego, USA). This method combines ARTIC multiplex PCR protocol with Illumina sequencing technology. Deep sequencing was performed on the NextSeq 500 platform (Illumina Inc., San Diego, CA, USA) using the NextSeq 500/550 High Output Reagent Cartridge v2, with 75 cycles and 36 bp paired-end reads. For bioinformatic analyses, all tools were run with default parameters unless otherwise specified. Quality control of the reads was performed using FastQC. Reads obtained were trimmed by Trimmomatic [15]. SARS-CoV-2 consensus sequences were obtained using iVar [16], and reads were then mapped to the reference sequence (Wuhan-Hu-1 [GenBank accession number NC_045512]) by Snippy [17].

Consensus sequences were submitted to the Pango COVID-19 lineage assigner [18] for lineage assignment. Obtained sequences were submitted to GISAID (Table 1). Sequences of the N gene were manually inspected and aligned with the reference sequence (Wuhan-Hu-1 [GenBank accession number NC_045512]) and N-positive B.1.177.75 sequences by MegAlign Pro (Lasergene; DNASTAR, Madison, WI, USA). All B.1.177.75 (*n* = 686) and B.1.177.8 (*n* = 1251) sequences available in GISAID (up to 15 April 2021) were downloaded and aligned using MAFFT implementing an FFT-NS-2 algorithm [19]. Alignments were manually inspected in Geneious Prime^®^ 2021.1.1 [20] and identical sequences were removed. A phylogenetic tree was estimated using IQTree with the Hasegawa-Kishino-Yano nucleotide substitution model and a gamma distributed rate variation among sites (HKY+Γ) as well as 1000 rounds of SH-like approximate likelihood ratio test (SH-aLRT) for branch support [21,22]. The N-gene from one of the +/+/- samples was used for a blastn search via the National Center for Biotechnology Information (NCBI) and the top 100 hits were added to the data set.

**Table 1.** Sampling and onset of symptoms date (day, month, year), cluster, GISAID virus name, *C_T_* for the ORF1ab, S-gene, N-gene, and N1.

Sampling date	Onset of symptoms	Epi Cluster	GISAID Sequence	ORF1ab	S-gene	N-gene	N-1
19/02/2021	17/02/2021	A	hCoV-19/Italy/ABR-IZSGC-101854/2021	21	22	>40	24
19/02/2021	19/02/2021	A	hCoV-19/Italy/ABR-IZSGC-101848/2021	19	20	>40	22
19/02/2021	18/02/2021	A	hCoV-19/Italy/ABR-IZSGC-101850/2021	19	21	>40	23
20/02/2021	19/02/2021	A	hCoV-19/Italy/ABR-IZSGC-102697/2021	20	22	>40	22
22/02/2021	20/02/2021	A	hCoV-19/Italy/ABR-IZSGC-109377/2021	21	21	>40	26
23/02/2021	19/02/2021	A	hCoV-19/Italy/ABR-IZSGC-109151/2021	17	17	>40	22
23/02/2021	18/02/2021	A	na	17	18	>40	na
23/02/2021	20/02/2021	A	hCoV-19/Italy/ABR-IZSGC-109144/2021	20	20	>40	23
23/02/2021	20/02/2021	A	hCoV-19/Italy/ABR-IZSGC-109140/2021	26	27	>40	29
26/02/2021	19/02/2021	A	hCoV-19/Italy/ABR-IZSGC-118434/2021	15	15	>40	25
27/02/2021	24/02/2021	B	hCoV-19/Italy/ABR-IZSGC-121069/2021	20	22	>40	25
03/03/2021	02/03/2021	B	hCoV-19/Italy/ABR-IZSGC-127721/2021	15	14	>40	25
03/03/2021	Asymptomatic case	B	hCoV-19/Italy/ABR-IZSGC-129620/2021	22	21	>40	31
03/03/2021	Asymptomatic case	B	hCoV-19/Italy/ABR-IZSGC-129622/2021	27	28	>40	31
03/03/2021	02/03/2021	B	hCoV-19/Italy/ABR-IZSGC-127720/2021	23	15	>40	32
05/03/2021	02/03/2021	B	hCoV-19/Italy/ABR-IZSGC-136239/2021	17	19	>40	na
05/03/2021	26/02/2021	B	hCoV-19/Italy/ABR-IZSGC-136241/2021	26	27	>40	na
01/03/2021	27/02/2021	na	hCoV-19/Italy/ABR-IZSGC-122959/2021	18	19	>40	21
04/03/2021	Asymptomatic case	na	hCoV-19/Italy/ABR-IZSGC-132139/2021	20	20	>40	na
06/03/2021	23/02/2021	na	na	27	28	>40	na

Notes: Na, not available. *C_T_* values ≥40 were considered negative according to the manufacturer’s instructions.

## Results

### SARS-CoV-2 positive samples showing the +/+/- pattern are circulating in central Italy

From 20 February to 6 March 2021, a total of 32,681 nasopharyngeal swab specimens were tested at IZSAM for SARS-CoV-2 RNA by means of the TaqPath COVID-19 CE-IVD RT-PCR Kit. A total of 5,277 swabs tested positive for SARS-CoV-2 in the study period (5277/32,681, 16.15%). Of these, 17/5277 (0.32%) showed a diagnostic pattern +/+/- for the ORF1ab, S and N genes respectively. *C_T_* values, sampling date and onset of symptoms related to these cases are showed in Table 1. These samples were collected from individuals living in three municipalities located in the province of Chieti: Torino di Sangro, Lanciano, and Paglieta.

### All N-negative SARS-CoV-2 strains belonged to the B.177.75 lineage

Fifteen of the 17 samples showing the +/+/- pattern were sequenced by NGS in addition to the early three strains for a total number of 18 sequenced strains. Total numbers of raw reads ranged from 687,884 to 5,668,968, with an average base quality score of 35.2. The numbers of mapped reads (151 nucleotides [nt] in length) ranged from 314,102 to 1,232,298, with coverage depth ranging from 1482X to 5359X. Consensus sequences were released on GISAID and accession numbers are showed in Table 1. These sequences were initially classified as B.1.177.8 via the Pango COVID-19 lineage assigner but were then updated to B.1.177.75 on 16 April 2021.

**Figure 1.**
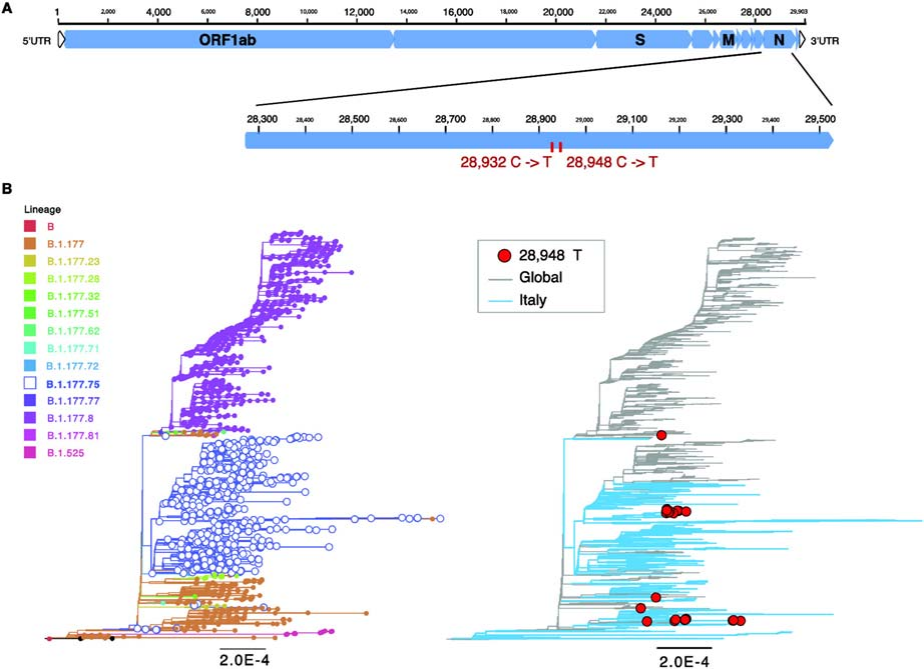
Genome organization of SARS-CoV-2 and variant specific mutations. (A) Shown is a schematic view of the full genome of SARS-CoV-2 (top) and the N gene enlarged (bottom) to simplify visualization of the two mutations C to T nucleotide substitutions at position 28,932 and 28,948 (ref accession NC_045512). (B) Maximum likelihood (ML) trees for SARS-CoV-2 full genomes (*n*=1858) including lineages B.1.177 (*n*=246), B.1.177.8 (*n*=910), B.1.177.75 (*n*=642), and others (*n*=62). Italian sequences (*n*=567) are shown in blue and global sequences in grey. Lineage specific mutations are indicated. Sequences containing mutant variant for 28,948 in red (right). Branch length indicates nucleotide substitutions per site. Tree is rooted at the reference sequence NC_045512.

N-gene sequences were manually inspected and compared to the original SARS-CoV-2 genome (NC_045512) and to reference sequences of lineage B.1.177.75 available on GISAID. Interestingly, a unique mutation, synonymous 28948C > T, was found in the N-negative B.1.177.75 strains. In addition, a non-synonymous mutation was present at position 28932C > T (N gene A220 V) which was shared by all B.1.177.75 sequences (Figure 1A). Thus, B.1.177.75 virus variants containing the 28932C > T mutation were diagnosed as positive for SARS-CoV-2 RNA with the canonical diagnostic +/+/+ pattern, while B.1.177.75 virus variants containing both the 28932C > T and 28948C > T mutation resulted in +/+/- for ORF1ab, S and N genes, respectively. Of note, N-negative B.1.177.75 tested positive when processed by 2019-nCoV_N1 whose primers and probe have a 100% match with a well-conserved portion of B.1.177.75 strains. *C_T_* values are also shown in Table 1.

Blastn search of the N gene from a sequence enclosing both 28932C > T and 28948C > T revealed the presence of five additional sequences with this very same mutation pair, all of which were from Switzerland. Notably, two of these Swiss sequences were obtained in October 2020. Phylogenetic evaluation showed the distinct separation between B.1.177.8 and B.1.177.75, with B.1.177 sequences being basal to both lineages (Figure 1B). Notably, none of the Italian sequences was classified as B.1.177.8 as per 16 April 2021. The T mutation at position 28,932 is present in all B.1.177.75 and B.1.177.8 sequences, as well as in some of the B.1.177 strains. The T mutation at position 28,948 was found in 38 sequences overall (B.1.177.75, *n* = 20; B.1.177, *n* = 14; other *n* = 4). This group includes also one sequence from Switzerland, which contains the wild-type C nucleotide at position 28,932 and the mutation T at position 28,948.

### Two apparently unrelated epidemiological clusters were evidenced

Information provided by the Local Health Authority highlighted the existence of two apparently unrelated clusters (cluster A and B), within the samples under investigation, plus three additional unrelated cases, all sustained by the N-negative SARS-CoV-2 strains of the B.1.177.75 lineage. In both clusters and all cases, clinical signs were generally mild and included fever, coughing, headache, and myalgia. They did not require hospitalization. Cluster A, identified from the first three samples collected on 19 February 2021, included 10 cases. It concerned two related families and two individuals living in adjacent houses. The first case of cluster A, a 3-year-old child, showed clinical signs on February 17, followed by his parents (February 18 and 19) and his older sister (February 20). Both children attended the same day-care in Torino di Sangro where several SARS-CoV-2 cases (nine cases in total, including the two in our study population) were detected starting from February 15. One of these cases was the teacher of the positive 3-year-old child. Interestingly, none of the other SARS-CoV-2 cases related to the day-care, including the teacher, showed the +/+/- pattern, as they were positive also for the N-gene. The rest of the cases belonging to cluster A showed clinical signs shortly after the first case, from February 18–20.

Cluster B grouped seven cases identified from February 27 to March 5. The cases, of which only five were symptomatic, belonged to two families living in Lanciano (Abruzzo region), linked by means of two teenagers attending the same class. Interestingly, two swab samples collected from scholars of the same school on March 2 and 3, respectively, showed the +/-/+ diagnostic pattern, a feature that, with the adopted molecular assay, is strongly suggestive of the B.1.1.7 lineage, which was widespread in the area [23,24]. Thus, the cluster B cases were not likely linked to those occurring at school.

**Figure 2.**
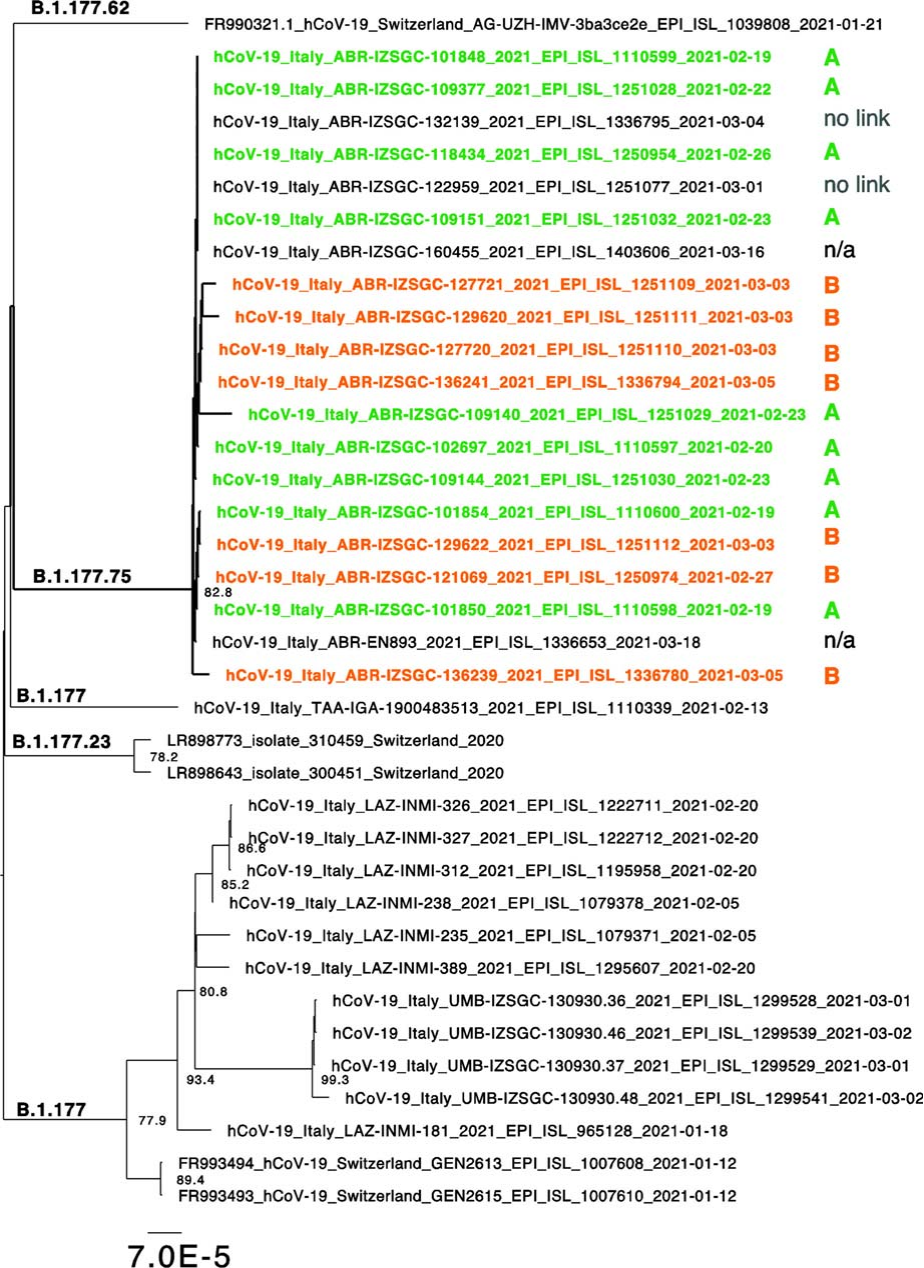
Sequence clusters for variants containing a T at position 28942. Extraction of the ML tree shown in Figure 1 for those sequences congaing the mutation leading to the +/+/- pattern. Lineages are indicated and sequences belonging to cluster A or B are coloured in orange a green, respectively. Branch length is indicative of nucleotide substitutions per site.

Phylogenetic analysis showed that all sequences form a distinct clade, and thus, are genetically related to each other (Figure 2). Additional three unrelated cases showing the +/+/- diagnostic pattern were identified at IZSAM during the survey period but only two were sequenced. Among these, one involved an individual who worked in the same town as cases of cluster A; the remaining two cases did not have any apparent connection to the other cases.

In addition, two additional sequences, downloaded from GISAID, are present in this clade. One (hCoV-19/Italy/ABR-IZSGC-160455/2021) was collected after the survey period, while the other (hCoV-19/Italy/ABR-EN893/2021) was processed by another diagnostic and sequencing hub in Abruzzo region. Unfortunately, we do not have any epidemiological information of these cases.

## Discussion

We describe here the circulation of B.1.177.75 strains, which are N-negative when processed with one of the most common kit for SARS-CoV-2 RNA diagnosis, the TaqPath COVID-19 CE-IVD RT-PCR Kit manufactured by Thermofisher. This phenomenon is likely due to the onset, in the B.1.177.75 lineage, of the synonymous 28948C > T mutation. Although we do not have any knowledge upon the nucleotide sequences of the primers and probe adopted by this kit, it is likely that this gene dropout only occurs when 28948C > T is coupled with 28932C > T, this latter present in all B.1.177.75 sequences available on public databases. Thus, 28948C > T is the sole mutation characterizing the N-negative B.1.177.75 samples with respect to N-positive strains belonging to the same lineage. It is known that few mismatches in the oligonucleotide binding region can affect the amplification efficiency, with prevention of any amplification when located in the very 3′ end of the primer(s) or on the middle of the TaqMan probe [25–27]. In a TaqMan assay for detection of rabies virus RNA, the number of sequence mismatches between gene-specific oligonucleotides and the target sequence significantly affected amplification and point mutations at the centre of the probe resulted in false-negative results through the prevention of probe binding and subsequent fluorescence [28]. Point mutations at positions 11–17 of the probe target site were found to yield false-negative results in a real-time RT-PCR assay for human respiratory syncytial virus [29].

Mutations 28948C > T and 28932C > T in the N gene are not novel within the plethora of mutations evidenced during SARS-CoV-2 evolution and adaptation to the human host. Indeed, they have been noticed, independently and regardless of the lineage, in SARS-CoV-2 sequences available on public databases. However, we were not able to quantity their presence across the GISAID database. GISAID currently holds ∼1.5 million sequence entries which is too much data to analyse. Although, GISAID has the option to search for specific mutations/variants, those found in this study are not on that list, suggesting their global prevalence is low.

B.1.177.75 is an offspring lineage of the major B.1.177 lineage (also known as *Spanish variant*), which emerged in summer 2020 and quickly became the dominant lineage in Europe during fall as a result of opening borders in summer 2020. The earliest detection of B.1.177.75 is 2 September 2020 (https://cov-lineages.org/lineages/lineage_B.1.177.75.html). Sequences of this lineage released by the IZSAM mainly originated from the province of Chieti, specifically from the municipalities of Torino di Sangro and Lanciano. During the period of observation described in this study (20 February 2021 to 6 March 2021) 17 N-negative swab samples were identified but only 15 were sequenced. The presence of both mutations has been evidenced in the genomic sequences obtained straight from the swab samples.

As we did not observe any +/+/- diagnostic pattern prior to 19 February 2021 and considering that IZSAM processes for SARS-CoV-2 RNA the vast majority (up to 65%) of swab samples of the entire Abruzzo region, we may speculate that mutation 28948C > T emerged with case 2021TE101854, a 3-year-old child who likely got infected from a B.1.177.75 N-positive individual, potentially including his teacher at daycare. This speculation relies on the fact that, epidemiologically, this is the most likely scenario as 2021TE101854 upstream connections did not show this mutation (being N-gene positive by real-time RT-PCR) and as this child was the first case to show symptoms in the cluster. The phylogeny for sequences containing the +/+/- pattern strongly supports a genetic connection and one common source for clusters A and B, as well as for the epidemiologically unrelated cases. Epidemiological connections are difficult to establish, particularly when young people are involved. Though, genetic comparison was useful in supporting a connection rather than suggesting the independent emergence of the same mutation in two apparently unrelated clusters. In addition, the lack of epidemiological link suggests the presence of additional cases (not sequences) containing the +/+/- pattern. Indeed, additional SARS-CoV-2 diagnostic hubs are active in Abruzzo region, thus we cannot exclude that other samples, harbouring this mutation, might have been tested somewhere else and potentially processed with different diagnostic kits which do not reveal this diagnostic pattern.

This manuscript has certainly some pitfalls. First, we were not able to sequence any of the N-positive cases connected to the N-negative clusters as a consequence of the fast turnaround of samples at the COVID-19 diagnostic hub of the IZSAM. These samples have been unfortunately discharged before epidemiological investigations were carried out. Second, we did not demonstrate that the combination of 28948C > T and 28932C > T is, with certainty, responsible for the observed N-gene dropout. In this regard, however, it is important to point out that, in support to our hypothesis, 28948C > T is the only mutation differentiating N-negative from N-positive B.1.177.75 samples and that, when N-negative samples are tested with a different N-based molecular test, all of them tested positive.

The number of infections caused by N-negative B.1.177.75 strains is overall limited and, importantly, these strains do not seem to cause more severe disease or more sustained transmission. However, the evidence of this strain endowed with this genomic hallmark has obvious consequences for molecular diagnosis as Thermofisher TaqPath COVID-19 CE-IVD RT-PCR Kit is one of the most used assays for SARS-CoV-2 diagnosis. Albeit limited, this evidence highlights the need for continuous surveillance, sharing of genomic data, which are indeed essential to update molecular tests, and the need for multiple genetic targets in nucleic acid amplification tests.

One of the most worrisome and currently widespread lineages, the B.1.1.7, is characterized by the S-gene dropout when tested with the same assay used in this study. This characteristic is related to an in-frame, 6-nucleotide deletion in the S gene, which is responsible for a 2-amino acid deletion at positions 69 and 70 of the spike protein (69–70del). This particular deletion has been observed in multiple distinct lineages besides B.1.1.7, notably in the mink cluster V lineage from Denmark and also in some B.1.177 strains. It cannot be ruled out that, as a consequence of convergent evolution or homologous recombination events, the 28948C > T and 28932C > T mutations may emerge in other lineages, including B.1.1.7, and that could lead to substantial diagnostic problems. Therefore, it remains essential to share genomic data if, when multi-target assays are used, novel diagnostic patterns are evidenced.
